# Multiple Parameters Beyond Lipid Binding Affinity Drive Cytotoxicity of Cholesterol-Dependent Cytolysins

**DOI:** 10.3390/toxins11010001

**Published:** 2018-12-21

**Authors:** Sucharit Ray, Roshan Thapa, Peter A. Keyel

**Affiliations:** Department of Biological Sciences, College of Arts and Sciences, Texas Tech University, Box 43131, Lubbock, TX 79409, USA; sucharit.ray@ttu.edu (S.R.); roshan.thapa@ttu.edu (R.T.)

**Keywords:** *Streptococcus pyogenes*, *Clostridium perfringens*, streptolysin, perfringolysin, membrane repair, pore-forming toxin, microvesicle shedding, intrinsic repair

## Abstract

The largest superfamily of bacterial virulence factors is pore-forming toxins (PFTs). PFTs are secreted by both pathogenic and non-pathogenic bacteria. PFTs sometimes kill or induce pro-pathogen signaling in mammalian cells, all primarily through plasma membrane perforation, though the parameters that determine these outcomes are unclear. Membrane binding, calcium influx, pore size, and membrane repair are factors that influence PFT cytotoxicity. To test the contribution of membrane binding to cytotoxicity and repair, we compared the closely related, similarly-sized PFTs Perfringolysin O (PFO) from *Clostridium perfringens* and Streptolysin O (SLO) from *Streptococcus pyogenes*. Cell death kinetics for PFO and SLO were different because PFO increased in cytotoxicity over time. We introduced known L3 loop mutations that swap binding affinity between toxins and measured hemolytic activity, nucleated cell death kinetics and membrane repair using viability assays, and live cell imaging. Altered hemolytic activity was directly proportional to toxin binding affinity. In contrast, L3 loop alterations reduced nucleated cell death, and they had limited effects on cytotoxicity kinetics and membrane repair. This suggests other toxin structural features, like oligomerization, drives these parameters. Overall, these findings suggest that repair mechanisms and toxin oligomerization add constraints beyond membrane binding on toxin evolution and activity against nucleated cells.

## 1. Introduction

The largest superfamily of virulence factors, utilized by many pathogenic bacteria, is the pore-forming toxins (PFTs). PFTs are generally classified by the secondary structure forming the pore, pore size, cellular target, and mechanism of binding. For example, cholesterol-dependent cytolysins (CDCs) are PFTs that are secreted from bacteria as soluble monomers, initially bind to cholesterol or specific proteins on the surface of the eukaryotic plasma membrane, oligomerize into a pre-pore, and insert in a cholesterol-dependent manner into the membrane as a β-barrel lined 20–30 nm pore [1]. Pore insertion can lead to many deleterious effects for the target cell, including cell death, enhanced infection, depolarization, and impairment of immune activation [2,3,4,5,6]. These effects can be lethal to the host, as is the case for necrotizing soft tissue infections (NSTIs). NSTIs, including gas gangrene that is caused by *Clostridium perfringens*, and necrotizing fasciitis caused by *Streptococcus pyogenes*, carry a mortality rate of 20–45% [2,7,8,9]. Key to these pathogens’ virulence are the CDCs Perfringolysin O (PFO) (*C. perfringens*), and Streptolysin O (SLO) (*S. pyogenes*) [2,10,11,12]. However, non-pathogenic and opportunistic bacteria also secrete CDCs and other PFTs with nonlethal outcomes [1,13,14]. The molecular mechanisms by which some PFTs drive lethal pathogenesis, while other PFTs do not [6,13], are poorly understood. 

Understanding the molecular mechanisms by which some PFTs, but not others, kill cells will help us to learn and improve on cellular mechanisms that preserve cell integrity, improve threat assessment of PFTs, and permit the elucidation of the properties of novel PFTs. Currently, PFT lethality is primarily determined based on physical aspects of the toxin, like pore-size, receptor target, binding affinity, and ion flux through the pore. However, host responses also limit cytotoxicity, suggesting that cellular responses must also be considered when determining how PFTs will affect cells.

One determinant that was proposed to account for differences in pathogenesis and host cell outcome following PFT intoxication is pore size. CDCs form large, heterogeneous pores ranging in size from 20–30 nm comprised of a variable number of subunits [1,15,16]. In contrast, small toxins, like aerolysin or hemolysin, form ≤5 nm pores with a well-defined stoichiometry [17,18]. Pore size is proposed to inversely correlate with membrane damage because aerolysin and hemolysin require hours for cellular recovery, whereas CDCs can be repaired in minutes [19,20,21,22,23,24]. Further support for this idea is the finding that narrowing the pore width on the PFT phobalysin P increased membrane permeability six hours after toxin challenge [21]. However, pore size is not a sufficient explanation for membrane damage, because increasing the pore width on the phobalysin P orthologue *Vibrio cholerae* cytolysin did not enhance recovery [21]. Pore size further fails to account for the finding that the small PFT sticholysin II is repaired with similar kinetics as the CDC listeriolysin O [20]. This suggests that pore size is not a good determinant of toxin cytotoxicity.

If pore size is not a good determinant, an alternative explanation for disparate host outcomes following PFT intoxication is the influx of second messengers, like Ca^2+^. For example, chelation of extracellular Ca^2+^ robustly decreases cell survival because it prevents some forms of membrane repair [19,23,24,25,26]. Consistent with this idea, membrane repair responses are triggered by small pores, like phobalysin P and sticholysin II, which promote Ca^2+^ influx, while these repair responses are not triggered by other small pores, like aerolysin, which may not promote Ca^2+^ influx [21,22,27,28]. However, Ca^2+^ influx is a complicated determinant, because it can influence both cell survival and cell death, suggesting that Ca^2+^ influx alone may not be sufficient to account for the differences observed. Since the extent of Ca^2+^ influx can lead to differential cellular effects [24,26,29,30], it is possible that the extent of Ca^2+^ influx determines cytotoxicity. However, for toxins that promote Ca^2+^ influx, the extent of influx is primarily a function of surface toxin pore concentration and extracellular Ca^2+^ concentration. For example, the CDC pneumolysin shows enhanced cytotoxicity at intermediate Ca^2+^ concentrations [30]. The extent of Ca^2+^ influx can be measured by fluorescently tagged annexins [24]. Thus, Ca^2+^ influx may help to determine survival, but it is not sufficient. 

A better determinant of survival might be measuring repair pathways downstream of Ca^2+^ influx that counteract PFT toxicity. Repair mechanisms downstream of Ca^2+^ influx include annexin recruitment, patch repair, and microvesicle shedding. Ca^2+^ influx activates many C2 domain proteins and annexins [29]. Ca^2+^ binding to annexin domains promote annexin translocation from the cytosol to the membrane when the intracellular Ca^2+^ concentration reaches a threshold concentration (~5 μM for Annexin A6 (ANXA6) [24]). Once on the membrane, annexins are hypothesized to form a barrier against membrane lesions [25,31,32,33,34,35]. Furthermore, C2 domain proteins are highly fusogenic, and promote the homo- and heterotypic fusion of vesicles and endolysosomes with the plasma membrane to seal off damaged areas following Ca^2+^ influx, in a process termed patch repair [29,36,37,38]. Finally, Ca^2+^ influx is important for the microvesicle shedding of PFTs [19,23,24,39]. PFTs are shed on microvesicles through protein-dependent shedding mechanisms, like the Endosomal Sorting Complexes Required for Transport (ESCRT)-mediated shedding [40,41], and/or through energy- and protein- independent, lipid-dependent mechanisms, like intrinsic repair [19,23]. Intrinsic repair is the spontaneous sequestration of toxins into small blebs, and subsequent shedding along with cellular proteins, including annexins [4,19,23,25]. Intrinsic repair is triggered by CDC oligomerization [19], suggesting that differences in oligomerization or toxin binding could alter repair responses. Although many CDCs share shedding responses [19,25], the rates of microvesicle shedding have not been compared across CDCs. This suggests that membrane repair might serve as one potential determinant of cytotoxicity.

Finally, one parameter that might integrate many of the above mechanisms is the determination of PFT binding target and affinity. Binding accessibility explains the difference in human and mouse sensitivity to the CDC pneumolysin O [42]. Furthermore, individual humans have a variable amount of accessible cholesterol [43], which could account for heterogeneous responses to CDCs. CDCs themselves may show a wide range of cell membrane cholesterol binding affinity, even amongst closely related CDCs, like PFO and SLO [1,44,45]. PFO and SLO both bind to cholesterol-rich membranes and the α-carbons of the membrane binding loops in the crystal structures of PFO and SLO overlap [1,44]. Although very similar, these CDCs exhibit different binding properties to cholesterol-rich membranes, with different consequences for cytotoxicity [44]. Notably, PFO binds more slowly to cells and cholesterol-containing liposomes than does SLO [44,45]. This difference is attributed to amino acid differences in membrane-binding loops near the cholesterol recognition motif [44,45]. For example, mutation of PFO D434 to Lys or Ser in the L3 loop increases the binding rate and affinity in both model liposomes and in cells, which make it similar to SLO [44,45]. Similarly, mutation of the homologous amino acid in SLO, S505, to Asp, decreases binding and switches this toxin to engage the membrane like PFO [44,45]. Interestingly, the cytotoxicity of PFO D434K is reduced in C2C12 myocytes due to a reduction in the extent of β-barrel insertion, whereas SLO S505D was reported to kill similarly to wild-type SLO [44]. Importantly, the cell death kinetics and membrane repair responses were not examined. Decoding the optimal toxin lipid environments may help us better understand the parameters that drive cytotoxicity.

Here, we took advantage of the similarities between two CDCs, PFO and SLO, to test the hypothesis that swapping residues in the L3 loop crucial for the extended binding interface would alter their overall cytotoxicity, cytotoxic kinetics and repair responses. We found a kinetic difference in the rate of cell death between wild-type PFO and SLO. We determined this was not due to assay conditions, cell type, nor extent of calcium influx. When we mutated key residues in the L3 loop, we found that these mutations changed the hemolytic activity as expected. Surprisingly, these mutations generally impaired cytotoxicity kinetics, and did not alter toxin-specific membrane repair kinetics. These findings suggest that membrane binding drives hemolytic activity, but other factors like oligomerization may drive membrane repair responses and impact cytotoxicity. We propose that binding and hemolytic activity are both insufficient to determine the sensitivity of nucleated cells to PFTs. Instead, repair and toxin oligomerization should also be considered. 

## 2. Results

### 2.1. PFO and SLO Kill Nucleated Cells with Different Kinetics

**Figure 1:**
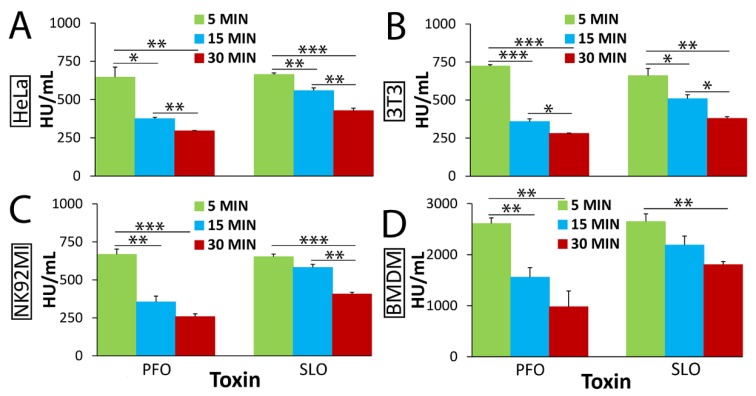
Perfringolysin O (PFO) and Streptolysin O (SLO) kill cells with different kinetics. (**A**) HeLa, (**B**) 3T3, (**C**) NK92MI or (**D**) Caspase 1/11^−/−^ bone-marrow derived macrophages (BMDM) were unchallenged or challenged for 5, 15 or 30 min with (**A**–**C**) 31-2000 HU/mL or (**D**) 125-8000 HU/mL PFO or SLO at 37 °C in Roswell Park Memorial Institute medium (RPMI) supplemented with 2 mM CaCl_2_ (RC) and 20 μg/mL propidium iodide (PI). The amount of toxin needed to kill 50% of the cells (EC_50_) at each time point was calculated by linear regression using the linear portion of the death curve. Graphs display the average EC_50_ ± SEM of five (HeLa, 3T3) or three (NK92MI, Caspase 1/11^−/−^ BMDM) independent experiments. * *p* < 0.05, ** *p* < 0.01, *** *p* < 0.001.

To test if the differences in membrane binding between PFO and SLO change the rate at which these PFTs kill nucleated cells, we challenged cells with equivalent hemolytic doses of PFO or SLO for 5 to 30 min and measured cytotoxicity by flow cytometry using propidium iodide (PI) uptake (Figure 1 and Supplemental Figure S1). We normalized toxin concentration by hemolytic dose instead of by mass because it was critical for successfully interpreting results and ruling out lot-to-lot variation in the specific hemolytic activity of recombinant toxins [23,46]. It was further important to normalize to hemolytic dose because it allowed us to specifically compare changes in membrane repair between different toxins and their lytic mutants, without interference from overall changes in hemolytic activity due to structural changes. We used these data to determine the toxin dose needed for 50% lysis of the cells (EC_50_) for each toxin at each time point by linear regression. Lower EC_50_ values represent increased toxicity and changes in EC_50_ values provide one measure of cell death kinetics. We measured cell death kinetics by comparing the EC_50_ determined at 5 min with the EC_50_ determined at 15 min or 30 min for both toxins. Both PFO and SLO had an EC_50_ of ~650 HU/mL in HeLa cells at 5 min (Figure 1A). While SLO showed a slight decrease in EC_50_ to 427 ± 17 HU/mL over 30 min, the EC_50_ for PFO decreased more than that in 15 min, and down to 295 ± 2 HU/mL by 30 min (Figure 1A). To control for any impurities in the toxin preparation, we challenged cells with an equivalent mass of the nonhemolytic SLO G398V/G399V “monomer-locked” (SLO ML) [19,47]. SLO ML can bind to the membrane, but has extreme defects in oligomerization, which prevents pre-pore and pore formation, and does not trigger membrane repair responses [19,47,48]. Consistent with previous results [19,47], SLO ML did not cause cytotoxicity at any time point observed (Supplemental Figure S1). To test if these temporal changes in EC_50_ were specific to HeLa cells, we next challenged a range of cell types including murine fibroblasts (3T3), a human natural killer cell line (NK92MI) and primary murine bone-marrow derived macrophages (BMDM) with CDCs (Figure 1B–D). To rule out any role for toxin-induced pyroptosis [15,49], we used BMDM from Caspase 1/11^−/−^ mice for all experiments involving BMDM. In 3T3 and NK92MI cells, we found that PFO increased in cytotoxicity over time. The EC_50_ for PFO rapidly decreased in 15 min, and dropped to ~275 HU/mL by 30 min (Figure 1B,C). In contrast, SLO decreased more slowly, reaching ~400 HU/mL by 30 min (Figure 1B,C) Casp1/11^−/−^ BMDM showed similar decrease from 2611 ± 112 HU/mL to 978 ± 310 for PFO and from 2650 ± 150 down to 1806 ± 60 HU/mL for SLO (Figure 1D). These results indicate that PFO and SLO kill multiple cell types with different kinetics, with PFO becoming more lethal over time than SLO. 

To further examine this phenotype, we first confirmed that it was not an artifact of our assay or of cell numbers. Our assay measures cell death using uptake of small fluorescent molecules, which might not faithfully report cell death. We compared the accuracy of our PI assay to MTT and LDH assays, which measure mitochondrial activity and the release of very large (>130 kDa) proteins, respectively (Supplemental Figure S2). As previously reported [23], the MTT assay showed similar degree and kinetics of cell death when compared to our flow cytometry assay (Supplemental Figure S2A). In contrast, the LDH assay showed the same trend, but underreported absolute cell death when compared to the other two cytotoxicity assays (Supplemental Figure S2B). These findings suggest that LDH release reports trends in cell death but does not accurately report the number of dead cells. We next controlled for the toxin concentration and number of cells used in our assay. When we varied toxin concentration, we observed changes in cell death from minimal, sublytic doses to highly lytic doses over a small (4–8 fold) range (Supplemental Figure S1). In contrast, when we varied the cell number, we observed no change in EC_50_ or cell death at cell numbers commonly used in assays for all cell types (Supplemental Figure S3). However, very large changes in cell number altered the EC_50_ and extent of cell death (Supplemental Figure S3A,B). These results indicate that the ratio of toxin to cells does not alter the extent of cell death under the standard assay conditions we use, suggesting that other parameters may control cell death.

### 2.2. SLO and PFO Kill Independently of Extracellular Calcium Concentration

**Figure 2:**
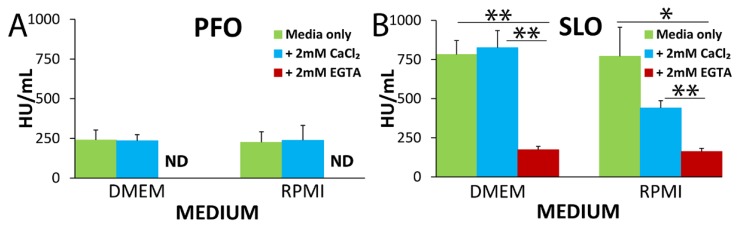
Variations in calcium influx do not account for changes in cytotoxicity. HeLa cells were unchallenged or challenged with 31–2000 HU/mL (**A**) PFO or (**B**) SLO in DMEM (1.8 mM Ca^2+^) or RPMI (0.42 mM Ca^2+^) with 20 µg/mL PI only or supplemented with either 2 mM CaCl_2_ or 2 mM EGTA for 30 (PFO) or 5 (SLO) min at 37 °C. The EC_50_ for each condition was calculated by linear regression using the linear portion of the death curve. The graphs display the average EC_50_ ± SEM of three independent experiments. ND indicates not determined due to high (<31 HU/mL) cytotoxicity. * *p* < 0.05, ** *p* < 0.01.

One important variable for PFT-induced cell death is calcium influx. The rate of Ca^2+^ influx is dependent on pore size, open pore concentration on the cell surface, and external Ca^2+^ concentration. We first tested the role of external Ca^2+^ concentration and culture medium across a range of toxin concentrations. In previous assays, we used Roswell Park Memorial Institute medium (RPMI) (0.42 mM Ca^2+^) supplemented with calcium to a final extracellular Ca^2+^ concentration of 2.42 mM to approximate physiological serum Ca^2+^ concentration. We tested whether changing the amount of extracellular calcium or if using a different media formulation (Dulbecco’s minimal essential medium (DMEM), 1.8 mM Ca^2+^) altered cytotoxicity. We challenged HeLa cells with PFO or SLO at similar doses in DMEM and RPMI alone or supplemented with 2 mM CaCl_2_ (Figure 2 and Supplemental Figure S4). In contrast to pneumolysin [30] and SLO (Figure 2), no effect of media or calcium concentrations between 0.42 mM and 3.8 mM was observed for PFO (Figure 2 and Supplemental Figure S4). The EC_50_ for SLO was lower for RPMI with Ca^2+^ than either RPMI or DMEM alone, or DMEM with Ca^2+^ (Figure 2, Supplemental Figure S4). The choice of media for cytotoxicity assays was not critical, because we observed similar results between DMEM and RPMI, though the combination of RPMI with Ca^2+^ improves toxicity for SLO. When we chelated the calcium in the medium using 2 mM EGTA, we found significant increases in the cellular sensitivity to PFO and SLO (Figure 2 and Supplemental Figure S4). Consistent with previous results [19,23,24,25,26], these results confirm that the membrane repair responses to CDCs are significantly diminished in the absence of calcium. Overall, these data indicate that the extracellular calcium concentration does not account for the difference in cell death that we observed between PFO and SLO. 

### 2.3. The L3 Loop Controls Both Rate and Extent of Cell Death

**Figure 3:**
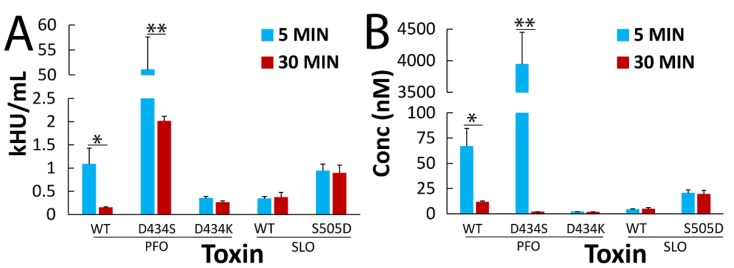
Changes in the cholesterol-dependent cytolysin (CDC) lipid-binding interface alter CDC cytotoxicity and kinetics. HeLa cells were unchallenged or challenged with 31–2000 HU/mL of either wild-type (WT) PFO or SLO, PFO D434S, PFO D434K, or SLO S505D in RC with 20 µg/mL PI for 5 or 30 min at 37 °C. The EC_50_ for each toxin and time point against HeLa cells was calculated by linear regression using the linear portion of the death curve and is displayed either in (**A**) HU/mL or (**B**) nM. The graphs display the average EC_50_ ± SEM of three independent experiments. * *p* < 0.05, ** *p* < 0.01.

Since both CDCs are similarly sized and differences in extracellular calcium concentration did not change cytotoxicity, we next tested whether CDC membrane binding alters cytotoxicity. We generated previously characterized [44,45] mutant variants of PFO and SLO with altered lipid binding. These CDCs have a single mutation in the L3 loop that swaps the lipid binding properties from PFO to SLO (PFO D434S/K) or from SLO to PFO (SLO S505D) [44,45]. PFO D434S and D434K have ~two-fold increased binding when compared to PFO, whereas SLO S505D has decreased binding as compared to SLO [44]. We found that all mutant toxins were hemolytic (Figure 3 and Supplemental Figure S5A), consistent with previous findings [44]. We challenged HeLa cells with wild-type PFO, PFO D434S, PFO D434K, wild-type SLO, or SLO S505D for 5 or 30 min (Figure 3). We found that PFO showed an eight-fold increase in EC_50_ over time, while PFO D434S showed a 25-fold increase (Figure 3A). In contrast to wild-type PFO, PFO D434K acted more similarly to SLO, in that it did not show a significant kinetic difference in cytotoxicity between 5 and 30 min (Figure 3A). Interestingly, PFO D434S was substantially less cytotoxic than wild-type PFO (Figure 3A). SLO S505D failed to introduce the predicted kinetic difference in cytotoxicity, but instead it reduced the overall cytotoxicity when compared to SLO (Figure 3A). We next determined whether the change in cytotoxicity was due to a decreased ability of the CDCs to perforate the cell, or due to upregulation of other cell survival mechanisms. To test this idea, we examined transiently permeabilized cells that survive the CDC challenge and remain metabolically active, identified as the PI^low^ population [15,19,23]. If cell permeabilization is decreased, we expect to observe a decrease in PI^low^ cells, while the upregulation of other cell survival mechanisms would show an increase in PI^low^ cells. When we examined the PI^low^ populations after mutant CDC challenge, we found no increase in this population (Supplemental Figure S5B). The comparison of the total permeability at a low (125 HU/mL) or high (1000 HU/mL) dose of toxin confirmed that the mutant toxins were overall less cytotoxic at equivalent hemolytic concentrations (Supplemental Figure S5C). Overall, we find that lipid engagement in the L3 loop is critical for the optimal cytotoxicity of CDCs in nucleated mammalian cells.

We observed apparent differences in cell death when compared to previous results [44], though these findings can be reconciled by considering normalization approaches. We measured nucleated cell death normalized to hemolytic activity instead of protein concentration, because it controlled for specific activity between toxin preparations and allowed for the comparison of membrane repair mechanisms that are not present in erythrocytes. We observed high hemolytic activity of lipid binding mutants, which is consistent with the reported increase in binding activity for PFO mutants [44,45]. Farrand et al. reported a two-fold increase in EC_50_ for PFO D434K over wild-type PFO in C2C12 myocytes [44]. However, we found that our EC_50_ values for wild-type PFO and PFO D434K were the opposite of theirs when determined by protein concentration (Figure 3B). Similarly, we found that our SLO was more potent and SLO S505D less potent than what they reported (Figure 3B and [44]). However, Farrand et al. also reported a two-fold decrease in hemolytic activity for PFO D434K over PFO at 30 min [44], which puts our PFO D434K findings in agreement with theirs when normalized for hemolytic activity, despite our use of different cell types. Although PFO D434K had similar cytotoxicity to SLO, we found a dramatic decrease in the cytotoxic activity of PFO D434S in HeLa cells (Figure 3). In contrast, PFO D434K showed a very similar EC_50_ to SLO (Figure 3). SLO S505D did not show a kinetic difference in HeLa cytotoxicity, but the EC_50_ at 5 min was similar to PFO. These data suggest that PFO D434K is similar to SLO, as predicted by the binding affinities. In contrast, SLO S505D shows similar initial cytotoxicity to PFO, but does not have the increase in cytotoxicity over time, like PFO. This implies that there are mechanisms beyond reduced binding that enhance PFO cytotoxicity over time. We attribute the differences in cytotoxic activity to membrane repair mechanisms that are present in HeLa cells that are absent in erythrocytes.

### 2.4. PFO Triggers Increased Microvesicle Shedding

If differences in cytotoxicity are due to membrane repair, this could account for differences in cell death kinetics. We tested the hypothesis that the toxin mutants show alterations in membrane repair using live cell imaging. To ensure that live cell imaging is comparable to flow cytometry, we tested whether multiple viability dyes or the adherence of cells altered the results. When we compared PI to other nuclear viability dyes YO-PRO, TO-PRO3, and DAPI; or the membrane-impermeant lipid-binding dye FM1-43X, we found that dye choice did not alter cell death measurements, and they stained similar cell populations (Supplementary Figure S6A,B). We next tested whether adherence altered cell death kinetics from CDCs. We found that the kinetic differences between PFO and SLO in suspension (Figure 1) were also observed when adherent cells were challenged with toxin (Supplementary Figure S6C). These data suggest that flow cytometry and live cell data should be comparable. 

**Figure 4:**
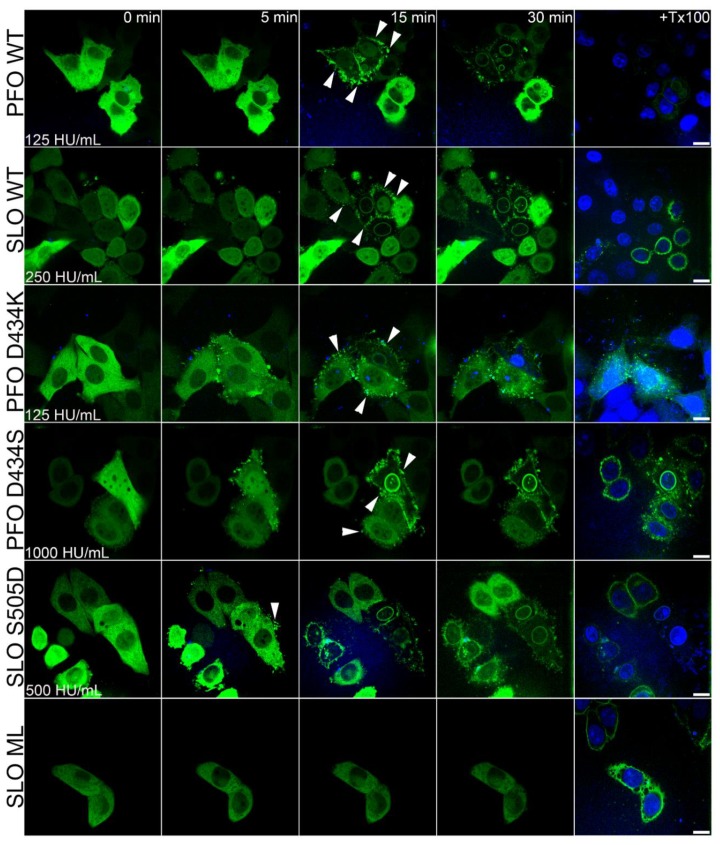
CDCs undergo different rates of Annexin A6-YFP (ANXA6-YFP) translocation. Annexin A6 YFP (green) transfected HeLa cells were challenged with the indicated toxins at the indicated sublytic concentrations in RPMI with 2 µg/mL TO-PRO3 (blue), 25 mM HEPES, pH 7.4, and 2 mM CaCl_2_. The cells were immediately imaged by confocal microscopy for ~45 min at 37 °C and then treated with 2% Triton-X (+ Tx100). ANXA6-YFP recruitment to the sites of plasma membrane damage from the cytosol is shown by arrowheads. The time following toxin addition is shown. Micrographs show representative, bleach-corrected images from six (SLO, PFO), five (SLO ML), or three (SLO S5050D, PFO D434S, PFO D434K) independent experiments. For each toxin, 12–15 toxin-cells were analyzed. Scale bar = 10 μm.

**Figure 5:**
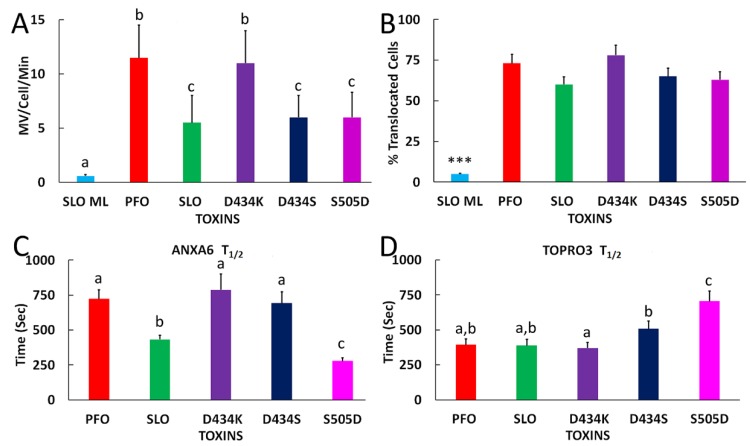
Membrane repair parameters are toxin specific. The live cell imaging performed in Figure. 4 was analyzed as follows: (**A**) Microvesicle shedding was manually counted and expressed as microvesicles shed/cell/min. (**B**) The fraction of cells showing ANXA6-YFP translocation was determined by measuring the percentage of cells with <80% ANXA-YFP intensity at 15 min. (**C**) ANXA6-YFP depletion or (**D**) TO-PRO3 uptake over time following toxin challenge was calculated from cells showing ANXA6-YFP translocation by determining the average intensity in a central region of the cell. The intensity was then normalized on a per cell basis, and these values averaged for each experiment. The time required to reach 50% (**C**) minimum or (**D**) maximum intensity (t_1/2_) was then determined and plotted for (**C**) ANXA6-YFP or (**D**) TO-PRO3. The graphs display the average ± SEM from six (SLO, PFO), five (SLO ML), or three (SLO S5050D, PFO D434S, PFO D434K) independent experiments. For each toxin, 12–15 toxin-cells were analyzed. Letters (a,b,c) represent statistically significant (*p* < 0.05) groups determined by repeated-measures ANOVA between groups. *** *p* < 0.001 compared to all other groups.

We then used live cell imaging to assess cellular membrane repair responses. Microvesicle shedding eliminates CDCs from the cell [19,23,25,50], so we used live cell imaging to quantitate microvesicle shedding in HeLa cells that were transfected with Annexin A6-YFP (ANXA6-YFP). We used ANXA6-YFP because it is a repair protein, translocates to the membrane following Ca^2+^ influx, and localizes to shed blebs [24]. We challenged HeLa cells with a sublytic toxin dose, imaged for 45 min in the presence of TO-PRO3 to mark and identify any dead cells, and then added Triton-X-100 at the end of the experiment to confirm maximum TO-PRO3 staining and ANXA6-YFP translocation. We found that all active toxins induced ANXA6-YFP translocation without TO-PRO3 accumulation, confirming our sublytic dose (Figure 4 and Supplemental Videos S1–S5). In response to toxin challenge, ANXA6-YFP was recruited first to specific sites on the plasma membrane (Figure 4). Later, translocation to the entire plasma membrane and nuclear membrane was observed (Figure 4). We did not observe ANXA6 cycling on and off the membrane, as has been reported for ANXA6 [24]. However, our results are within the range of reported ANXA6-YFP behaviors [25]. We measured ANXA6-YFP shedding. We found that both wild-type and lipid-binding mutant toxins induced microvesicle shedding (Figure 5A, Supplemental Videos S1–S5, Supplemental Figure S7A). Interestingly, we found that both PFO and PFO D434K showed a significant increase in the number of microvesicles shed when compared to the other active toxins (Figure 5A), though the total fluorescence of released ANXA-YFP was less significant (Supplemental Figure S7). PFO D434S, SLO, and SLO S505D showed a similar extent of shedding, while SLO ML only induced background levels of shedding (Figure 5A, Supplemental Video S6, Supplemental Figure S5). The differences in shedding could be due to the different extent of permeabilization by toxin. To test this possibility, we measured the proportion of cells that were permeabilized as measured by ANXA6-YFP translocation. We measured ANXA6-YFP translocation to the membrane by depletion rather than accumulation on the membrane due to cell motion in the periphery. Although this method might measure ANXA6-YFP leakage from permeabilized cells, previous work showed that ANXA6 remains membrane bound, even in fully permeabilized cells [24]. 

We found that all active toxins induced ANXA6-YFP translocation in similar proportions at their respective sublytic doses, suggesting that the extent of permeabilization was consistent across toxins (Figure 5B). In contrast, SLO ML failed to induce any translocation (Figure 5B). The lack of shedding or ANXA6-YFP translocation by SLO ML confirms that shedding is an active response to toxin, as previously reported [4,15,19,23,24,25,40,46,50,51]. Overall, these data suggest that shedding is independent of binding affinity.

We next compared the rate of ANXA6-YFP depletion from the cytosol as it translocates to the plasma membrane. We found that the rate of translocation was similar for all PFO mutants (Supplemental Figure S5B). In contrast, SLO induced translocation at a faster initial rate, but it induced a lower overall extent of translocation (Figure 5C). We found that the t_1/2_ remained ~700 s for all PFO mutants (Figure 5C). This suggests that the intracellular Ca^2+^ concentration may not elevate to the same extent in cells that are challenged with SLO. In contrast, SLO S505D induced translocation faster than all other toxins, and the extent of translocation was similar to PFO (Figure 5C). SLO ML did not induce substantial ANXA6-YFP translocation over time until the addition of Triton-X-100, indicating that ANXA6-YFP depletion was not due to photobleaching or phototoxicity (Figure 5C). These findings show that SLO S505D acts faster than wild-type PFO, as measured by ANXA6-YFP depletion.

ANXA6-YFP depletion reports calcium influx >5 µM [24], but it does not measure viability or dye ingress. We next analyzed TO-PRO3 nuclear staining because cells may take up small amounts of dye without dying when they are transiently permeabilized (Supplemental Figure S5C and [23]). When compared to lysis with Triton-X-100 at the end of the experiment, cells that were treated with sublytic doses of toxin gradually took up small amounts of TO-PRO3, except for SLO ML, which did not promote TO-PRO3 uptake (Supplemental Figure S5C). When we calculated the t_1/2_ for TO-PRO3 uptake, we found it to be equal for both PFO and SLO (Figure 5D). Interestingly, SLO S505D, which shows reduced cytotoxicity, displayed delayed TO-PRO3 uptake, while PFO D434S trended slower (Figure 5D). We then compiled all of these results to determine how each lipid binding mutant performed relative to the parental wild type toxins (Table 1). For example, PFO D434K had SLO-like membrane binding, hemolytic activity, kinetic difference, EC_50_ (measured in HU/mL), but PFO-like shedding, ANXA6 translocation, ANXA6 t_1/2_, and TOPRO3 t_1/2_ (Table 1). In some cases, such as the nM EC_50_, the mutant toxins behaved differently from both parental toxins (Table 1). Overall, these data suggest that lipid binding determines hemolytic activity, but cytotoxicity and extent of membrane repair are not dependent on lipid binding.

## 3. Discussion

Here, we examined the consequences of modifying the lipid-binding interface of cholesterol-dependent cytolysins for cytotoxicity and repair using the two closely related toxins, PFO and SLO, and found that binding affinity is not sufficient to drive cytotoxicity. PFO showed an increase in cytotoxicity over time, whereas SLO does not. We tested the hypothesis that this kinetic difference was due to binding affinity. We found that increasing toxins’ binding affinity also increased their hemolytic activity as expected, but surprisingly it had limited or deleterious effects on changes to cytotoxic activity and on cell death kinetics in nucleated cells. Similarly, membrane repair, as measured by microvesicle shedding kinetics and the rate of Annexin A6 translocation, was not substantially changed by alteration of the lipid binding surface. Overall, these findings suggest that lipid binding determines toxicity in cells with limited repair capacities, but it is not sufficient to determine the toxicity in cells with active repair mechanisms. In these cells, oligomerization and repair mechanisms may add additional constraints on toxin evolution and activity.

We found that cell death kinetics were different between two very similar CDCs, PFO, and SLO. We tested several hypotheses concerning the mechanism of this kinetic difference. We found that this kinetic difference was independent of cell number, cell type, or the amount of extracellular calcium. Both PFO and SLO form variably sized pores of 20–30 nm [1,15,16], suggesting that pore size does not account for changes in cytotoxicity. Importantly, the PFO D434K mutation does not alter pore size [44], yet PFO D434K behaved more like SLO than PFO for cell death kinetics. This finding provides further evidence that pore size does not drive the kinetic difference in cytotoxicity. Although other studies on toxin size, Ca^2+^ influx, and toxicity used smaller toxins [20,21,28], our findings support the idea [20] that pore size does not adequately determine cytotoxicity.

To better determine cytotoxicity, it is necessary to consider additional parameters, like membrane binding. We tested the impact of membrane binding on cytotoxicity using well-defined mutations in the L3 loop. Interestingly, we found that alterations in the L3 loop of CDCs changed both hemolytic activity and short-term (5 min) cytotoxic activity in nucleated cells as expected, but failed to alter sustained (30 min) cytotoxic activity in nucleated cells. PFO D434K, which increases binding similar to SLO, showed SLO-like cytotoxicity and hemolytic activity, whereas D434S only increased hemolytic activity. The SLO S505D mutation, which reduces binding to that of PFO, also reduced the initial cytotoxic activity and hemolytic activity to activities that are similar to PFO. These data suggest that binding changes driven by L3 loop mutagenesis successfully swap hemolysis and initial cytotoxicity.

Although binding affinity was proportional to hemolytic activity and initial cytotoxicity, it did not change the kinetics of cell death. In some cases, it greatly reduced the cytotoxicity of the domain swapped toxins. We found that reducing the binding affinity of SLO by introducing the analogous PFO mutation decreased its sustained cytotoxicity. Similarly, PFO D434S, which places the analogous residue from SLO into PFO [44,45], dramatically reduced all cytotoxicity, despite a high hemolytic activity and improved binding. Farrand et al. found that PFO D434K has reduced β-barrel insertion, which led them to propose the idea that the L3 loop helps target the CDC to membrane microdomains that better sustain pore insertion [44]. Consistent with that idea, we found that at 30 min, PFO was more cytotoxic than PFO D434K, though we note that PFO D434K remained a very potent toxin. We found that PFO D434K had similar cytotoxicity to SLO. Neither PFO D434K nor SLO shared the kinetic difference in cytotoxicity observed for PFO. It is not immediately clear how increased short-term cytotoxicity, but decreased sustained cytotoxicity, would result from reduced insertion of the CDC β-barrel. Since the extent of β-barrel insertion was measured after a 4 °C incubation step at one time point [44], both SLO and PFO D434K could show a reduced overall extent of β-barrel insertion, but still insert the β-barrel faster than PFO. In contrast, PFO might show very little β-barrel insertion at early time points, but increased insertion at later time points. This interpretation is consistent with the apparent speed versus quality difference that was observed between SLO and PFO. It is also consistent with the idea that membrane microdomains variably support pore insertion. PFO might take more time to find the optimal membrane microdomain for cytotoxicity, which would be facilitated by weaker binding, whereas the higher binding of SLO might not permit sufficient time to find the optimal membrane microdomain for cytotoxicity. PFO D434K acts similarly to SLO in this regard because the binding is increased, which does not give the toxin time to find the optimal binding target prior to insertion. This supports the idea [44] that CDCs use binding affinity to optimize trade-offs between speed and quality. However, reducing β-barrel insertion could reduce cytotoxicity via membrane repair. A reduction in β-barrel insertion would increase the extent of oligomers on the cell surface, which we have previously shown stimulate intrinsic repair [19].

To examine the relationship between membrane repair, cytotoxicity, and CDC binding affinity, we used ANXA6-YFP to compare approximate Ca^2+^ concentrations and two indicators of membrane repair, microvesicle shedding and ANXA6-YFP translocation to the membrane, between PFO, SLO and the lipid binding mutants. Interestingly, all modifications of the L3 loop generally drove the total Ca^2+^ influx to be more similar to PFO, as measured by the maximal extent of ANXA6-YFP translocation. However, alterations in the L3 loop did not change the rate of ANXA6-YFP translocation from that of the parental toxins. We interpret these findings as support for the idea that lipid-dependent repair mechanisms [19,23] can rapidly act to reduce or limit cytotoxicity prior to the engagement of protein dependent mechanisms. However, it is also possible that localized Ca^2+^ influx provokes patch repair mechanisms that slow and/or block continued Ca^2+^ influx prior to the recruitment of ANXA6-YFP to the membrane. It is also possible that intracellular Ca^2+^ stores are differentially regulated by PFO and SLO. For pneumolysin, low (1 mM) extracellular Ca^2+^ concentrations increased cytotoxicity [30], distinct from both of the toxins tested here. Since the pores are very large for these toxins and binding did not change Ca^2+^ influx, other factors must govern both extent of Ca^2+^ influx, and overall outcomes. We speculate this may be due to microdomain localization of each toxin, and/or lipid remodeling during repair. Either of these factors could lead to differences in signal transduction downstream of Ca^2+^ influx. Future work is needed to directly measure Ca^2+^ levels with indicator dyes, dissect the relative contributions of patch repair, annexin recruitment, and intrinsic repair to cell survival.

Interestingly, changes to binding affinity did not change microvesicle shedding kinetics. We found that PFO exhibited a greater rate of shedding than SLO. However, alterations to binding affinity through mutations in the L3 loop, which is predicted to alter the membrane microenvironment of the toxin, did not change the rate of shedding for PFO D434K and SLO S505D. This suggests that different regions or activities of the toxin may drive shedding, such as rate or efficiency of toxin oligomerization. It is also possible that there are lipid-binding determinants beyond the cholesterol-recognition motif and L3 loop that drive membrane repair responses. However, it is important to note that we did not measure the total number of pores on each shed bleb. It is possible that PFO is less efficiently loaded on blebs, so a greater shedding rate is necessary to yield the same degree of repair. It is also possible that inactive oligomers that are present in the toxin preparation alter repair. For example, we and others have previously shown that both active pores and inactive CDC oligomers are shed on microvesicles following SLO, PFO, and pneumolysin challenge [19,23,52]. We further limited our analysis to ANXA6-YFP^+^ membrane blebs. We cannot rule out shedding of blebs with ANXA6-YFP levels below our limit of detection, which likely occur, and may or may not be equivalent between PFO and SLO. Finally, we did not examine whether shedding occurred more frequently at earlier versus later time points because the detection of microvesicle shedding by live cell imaging is challenging. Analysis of total fluorescence from shed vesicles suggests that some of these factors may be involved, because the differences were not as pronounced in this assay. Future work is needed to better characterize shedding dynamics. Overall, we found that membrane repair is triggered by toxin-specific determinants beyond the lipid binding controlled by the L3 loop. 

Surprisingly, we found that two mutations that cause similar increases in PFO binding affinity had dramatically different cytotoxicity and repair profiles. When we compared PFO D434K with PFO D434S, we found that they shared a high hemolytic activity and ANXA6-YFP recruitment rate, but they differed in cytotoxicity kinetics, cytotoxic activity, shedding rate, and TO-PRO3 uptake. These differences highlight the fact that there remains much to be learned about toxin-membrane interactions, and that no single parameter adequately explains cytotoxicity. Instead, multiple parameters are needed to model cytotoxicity and cell survival.

Finally, while CDC cytotoxicity is the prime consideration for trade-offs between speed and quality, other virulence factors may influence these trade-offs because they cooperate with SLO and PFO. SLO, but not PFO, can induce cytosol-mediated translocation of the NAD^+^ glycohydrolase Spn [53,54]. The interaction of Spn with SLO can modify SLO membrane binding properties [55]. These interactions could place membrane localization and binding constraints on SLO that are not present for PFO. Similarly, while PFO is important for the virulence of gas gangrene [2], α-toxin, a zinc metallophospholipase, is critical for gas gangrene [2,56]. It is possible that α-toxin modifies the membrane to catalyze the rate or extent of PFO binding by generating membrane microdomains that are favorable to PFO. PFO binding is limited by sphingomyelin [57], which is cleaved by α-toxin, as is phosphatidylcholine [58]. Future work is needed to understand the cooperative effects of CDCs with other bacterial toxins and how they synergize in lethal diseases, like gas gangrene, necrotizing fasciitis, and septic cardiomyopathy.

## 4. Materials and Methods

### 4.1. Reagents

All reagents were from Thermofisher Scientific (Waltham, MA, USA), unless otherwise noted. Annexin A6-YFP was a generous gift from Annette Draeger (University of Bern, Bern, Switzerland) [24]. The pBAD-gIII plasmid encoding His-tagged SLO was a kind gift from Michael Caparon (Washington University in St. Louis, MO, USA) [47]. Cysteine-less His-tagged PFO in pET22 was a generous gift from Rodney Tweten (University of Oklahoma Health Sciences Center, Oklahoma City, OK, USA) [59]. Monomer-locked (G398V/G399V) SLO was previously described [19]. Lipid-binding mutants were introduced into SLO (SLO S505D) and PFO (PFO D434S and D434K) using Quikchange PCR. Primer sequences are available upon request. Propidium iodide (PI) (Lot # MKCB0899V) and DAPI (Cat # D9542) were from Sigma (St. Louis, MO, USA).

### 4.2. Mice

All mice were housed and maintained according to IACUC standards, adhering to the Guide for the Care and Use of Laboratory Animals (8th edition, NRC 2011) for animal husbandry. Animal use was approved by the Texas Tech University Animal Care and Use Committee, with protocol numbers 16090-10 and 16092-10, starting on 10/10/16 and 10/4/16, respectively. Caspase 1/11^−/−^ mice on the C57BL/6 background were purchased from the Jackson Laboratory (Bar Harbor, ME, USA) (stock # 016621) and bred in-house. Mice of both genders aged 6–15 weeks were used to prepare BMDM. Sample size was determined as the minimum number of mice needed to provide enough bone marrow for experiments. Consequently, no randomization or blinding was needed. Mice were sacrificed by asphyxiation through the controlled flow of pure CO_2_, followed by cervical dislocation.

### 4.3. Cell Culture

All cell lines were maintained at 37 °C, 5% CO_2_. HeLa cells (ATCC (Manassas, VA, USA) CCL-2) were cultured in DMEM (Corning, Corning, NY, USA) supplemented with 10% Equafetal bovine serum (Atlas Biologicals, Fort Collins, CO, USA) and 1× l-glutamine (D10). 3T3 cells (ATCC, CRL-1658) were cultured in D10 supplemented with 1 mM sodium pyruvate (Corning) and 1× non-essential amino acids (GE Healthcare, Pittsburgh, PA, USA). NK92MI cells (ATCC, CRL-2408) were cultured in Alpha MEM (GE Healthcare) supplemented with 10% Equafetal bovine serum, 1× l-glutamine, 0.2 mM myo-inositol, and 0.02 mM folic acid. Caspase 1/11^−/−^ macrophages were isolated from C57BL/6 mouse bone marrow and cultured as previously described [4]. BMDM were differentiated for 7–21 days prior to experiments in DMEM that was supplemented with 30% L929 cell supernatant, 20% premium fetal calf serum (VWR Seradigm, Radnor, PA, USA), 1 mM sodium pyruvate, and 1× l-glutamine.

### 4.4. Recombinant Toxins

Toxins were induced and purified as previously described [19,46]. Toxins were induced with 0.2% arabinose (SLO and SLO S505D), or 0.2 mM IPTG (PFO, PFO D434S, and PFO D434K) for 3 h at room temperature and purified using Nickel-NTA beads. Protein concentration was determined by Bradford Assay while hemolytic activity was determined as previously described [19,46], except human red blood cells (Zen Bio, Research Triangle Park, NC, USA) were used instead of sheep. One hemolytic unit is defined as the amount of toxin that is required to lyse 50% of a 2% human red blood cell solution in 30 min at 37 °C in 2 mM CaCl_2_, 10 mM HEPES, and 0.3% BSA in PBS. The EC_50_, specific activity and protein concentrations for each active toxin is listed in Table 2. Pore- and oligomerization-deficient SLO monomer-locked toxin had a specific activity of <10 HU/mg and was used at a mass equivalent to wild-type SLO. The sublytic dose was defined as the highest toxin concentration that killed <20% of target cells. For HeLa cells, the sublytic dose used was 1000 HU/mL for PFO D434S, 125 HU/mL for PFO D434K, and 250HU/mL for SLO S505D. 

### 4.5. Flow Cytometry Cytotoxicity Assay

Cytotoxicity was assessed as described [23]. Briefly, 1 × 10^5^ cells were challenged in suspension with various concentrations of CDC for 5 or 30 min at 37 °C in RPMI supplemented with 2 mM CaCl_2_ (RC) and 20 μg/mL PI and analyzed on a 4-laser Attune Nxt flow cytometer. Assay variations included changes to the incubation time, the cell number (5 × 10^4^, 10 × 10^4^ or 20 × 10^4^ cells), the media (RPMI or DMEM alone, with 2 mM CaCl_2_ or with 2 mM EGTA instead of CaCl_2_), the viability dye (2 μg/mL YO-PRO, TO-PRO3, FM1−43X or 0.5 μg/mL DAPI), or challenging plate-bound cells with toxin prior to harvest. For analysis, debris was gated out and the percentage of cells with high dye fluorescence (2–3 log shift) (dye high), low dye fluorescence (~1 log shift) (dye low), or background dye fluorescence (dye negative) was quantified. Both dye negative and dye low populations remain metabolically active (Supplemental Figure S2 and [23]), showing that only the dye high population are dead cells. Specific lysis was calculated as follows: % Specific Lysis = (% Dye High^Experimental^ − % Dye High^Control^)/(100 − % Dye High^Control^) × 100. Transiently permeabilized cells were calculated similarly, using dye low instead of dye high populations. The EC_50_ was defined as the toxin concentration needed to kill 50% of HeLa cells, and determined by regression of the linear portion of the kill curve using Excel (Microsoft, Redmond, WA, USA).

### 4.6. MTT/LDH Assays

HeLa cells were harvested and resuspended at 1 × 10^5^ cells per well in a 96 well V bottom plate in DMEM (without Phenol Red) supplemented with 2 mM CaCl_2_. Toxins were diluted in DMEM according to hemolytic activity (wild-type toxins) or equivalent mass (SLO ML), serially diluted two-fold, added to wells containing cells, and incubated for 5, 15, or 30 min at 37 °C. Triton was added to a final concentration of 0.5% to four wells containing cells as a positive control for maximum LDH release and incubated for 30 min. After incubation, cells were centrifuged at 1200× *g* for 5 min at 4 °C to pellet cells. Cell pellets were used for the 3-(4,5-dimethylthiazol-2-yl)-2,5-diphenyltetrazolium bromide (MTT) assay, while supernatants were assayed for lactose dehydrogenase (LDH) activity. For the MTT assay, cells were resuspended in 1.1 mM MTT assay reagent in DMEM without Phenol Red and incubated at 37 °C for 4 h. Formazan was dissolved in SDS-HCl at 37 °C overnight and absorbance was measured at 570 nm using a plate reader (Bio-Tek, Winooski, VT, USA). The % Viable cells was determined as follows: % Viable = (Sample − Background)/(Control − Background) × 100. Then, % Specific Lysis was calculated as 100% − Viable. For the LDH assay, cell supernatants were assayed per the manufacturer’s instructions. % LDH release was calculated as (Sample − Background)/ (Maximum LDH release − Background) × 100.

### 4.7. Live Cell Imaging

HeLa cells were plated at 2 × 10^5^ cells per 35 mm glass bottom dish (MatTek, Ashland, MA, USA) and transfected with 500 ng Annexin A6 YFP using Lipofectamine2000 two days prior to imaging. The transfection efficiency was 85%. Transfected cells were challenged with a sublytic CDC dose and imaged at 37 °C in RPMI, 25 mM HEPES pH 7.4, and 2 mM CaCl_2_ with 2 µg/mL TO-PRO3 for 45 min using a Yokogawa CSU-X spinning disc confocal microscope (Intelligent Imaging Innovation, Denver, CO, USA). ANXA6-YFP was excited using a 488 nm laser line, while TO-PRO3 was excited with a 640 nm laser line. Fluorescence was collected using a 60×, 1.49 NA oil immersion objective and recorded using an Evolve 512 EMCCD camera (Photometrics, Tucson, AZ, USA) at 1–3 sec/frame. After 45 min, an equal volume of 2% Triton-X-100 was added to give a final concentration of 1%. The total number of ANXA6-YFP^+^ microvesicles released was counted manually using every second frame and was expressed as number of microvesicles/number of cells/minute. The percentage of cells showing ANXA6-YFP translocation was determined by comparing the ANXA6-YFP intensity at 15 min to the intensity 1 min after toxin addition. If these values were below 80% of the initial value, cells were considered to show ANXA6-YFP translocation. In cells showing ANXA6-YFP translocation, the extent of ANXA6-YFP depletion from cells was assessed by measuring ANXA6-YFP intensity from the middle of the cell over time by plotting Z profiles in ImageJ (NIH, Bethesda, MD, USA). TO-PRO3 uptake was determined in ANXA6-YFP translocated cells by plotting the Z profile of a nuclear subsample over time. For both ANXA6-YFP and TO-PRO3, the data were normalized to the integrated intensity of the brightest point. To calculate t_1/2_ for Annexin depletion or TO-PRO3 uptake, the time at which the intensity was half-maximal was determined using the starting intensity and the average intensity of the last 4 min prior to Triton-X-100 addition. From the images, 12–15 cells were analyzed from at least three independent experiments and graphed using Microsoft Excel. For display (but not analysis), bleach correction for ANXA6-YFP was performed with histogram matching using Fiji (NIH, Bethesda, MD, USA). TIFF image sequences were exported, split into individual monochromatic red, green, and blue channels. The green channel was bleach corrected by histogram matching followed by a median pass filter. The monochromatic images were then merged to form RGB TIFFs, time-stamped, annotated, and exported as AVIs for supplementary videos.

### 4.8. AnnexinA6-YFP Fluorescence Quantification

HeLa cells were plated at 6 x 10^4^ cells per well in 24 well plates and transfected with 500 ng Annexin A6 YFP using Lipofectamine2000 two days prior to quantification. Transfected cells were challenged with a sublytic CDC dose and incubated at 37 °C in DMEM without Phenol Red, 25 mM HEPES pH 7.4 and 2 mM CaCl_2_ for 45 min. Supernatants were transferred to a 96 well flat clear bottom black walled plate and they were quantified using a Bio-Tek FL600 fluorescence plate reader at 530/25 nm wavelength with 125 sensitivity. Fluorescence intensity was expressed as Arbitrary Units (AU) after background subtraction. 

### 4.9. Statistics

Prism 5.0 (GraphPad, La Jolla, CA, USA) or Excel were used for statistical analysis. Data are represented as mean ± SEM as indicated. The EC_50_ for toxins was calculated by linear regression using the linear portion of the death curve. Statistical significance was determined by one-way ANOVA or repeated measures ANOVA; *p* < 0.05 was considered to be statistically significant. Graphs were generated in Excel and Photoshop (Adobe, San Jose, CA, USA).

## Figures and Tables

**Table 1:** Summary of L3 loop CDC mutant toxin behavior based on multiple parameters.

Parameter	PFO D434K	PFO D434S	SLO S505D
Membrane Binding	SLO	SLO	PFO
Hemolytic Activity	SLO	SLO	PFO
Kinetic Difference	SLO	PFO	SLO
EC_50_ HU 5 min	SLO	higher than both	PFO
EC_50_ HU 30 min	SLO	higher than both	higher than both
EC_50_ nM 5 min	lower than both	higher than both	intermediate
EC_50_ nM 30 min	lower than both	lower than both	higher than both
Shedding Rate	PFO	SLO	SLO
ANXA6 Max Translocation	PFO	PFO	PFO
ANXA6 t_1/2_	PFO	PFO	SLO
TOPRO3 t_1/2_	PFO	slower than both	slower than both

**Table 2:** Lytic parameters of toxins used in this study.

Toxin	EC_50_ (HU/mL)	EC_50_ (nM)	*p*-Value	Hemolytic Activity (HU/mL)	Protein Conc. (mg/mL)	Specific Activity (HU/mg)
SLO WT 5 min	339 ± 47.8	4.299 ± 0.606	ns	1.6 × 10^6^	1.4	1.14 × 10^6^
SLO WT 30 min	367 ± 107	4.649 ± 1.358				
SLO S505D 5 min	938 ± 148	20.397 ± 3.226	ns	1.6 × 10^6^	2.4	6.7 × 10^5^
SLO S505D 30 min	889 ± 176	19.321 ± 3.836				
PFO WT 5 min	1083 ± 345	63.752 ± 20.293	0.03616	2.56 × 10^6^	10.4	2.5 × 10^5^
PFO WT 30 min	148 ± 17.7	8.687 ± 1.043				
PFO D434S 5 min	51049 ± 6557	3942 ± 506.4	0.00147	2.56 × 10^7^	1.4	1.83 × 10^7^
PFO D434S 30 min	2008 ± 108	1.591 ± 0.086				
PFO D434K 5 min	347 ± 41.0	1.888 ± 0.223	ns	1.6 × 10^6^	0.6	2.7 × 10^6^
PFO D434K 30 min	257 ± 38.3	1.394 ±.208				

## Supplementary Materials

The following are available online at https://zenodo.org/record/2474310#.XBywssQRWUl: Figure S1: PFO and SLO kill cells with different kinetics over multiple concentrations. Figure S2: LDH release does not report number of dead cells. Figure S3: CDC cytotoxicity is independent of cell number within common assay ranges. Figure S4: Medium base and variations in calcium influx do not account for changes in cytotoxicity. Figure S5: Changes in CDC lipid-binding interface alter CDC cytotoxicity and kinetics. Figure S6: Dye choice and plastic adherence minimally impact cytotoxicity assay. Figure S7: CDCs induce different repair responses. Video S1: Repair induced by PFO. HeLa cells transfected with ANXA6-YFP (green) were challenged with 125 HU/mL wild-type PFO in the presence of 2 μg/mL TO-PRO3 (blue) and imaged at 37 °C by live cell confocal imaging at 1 frame/second. Triton-X-100 was added at the end as a positive control for cell permeabilization. Images were bleach-corrected by histogram matching. Time shows min:sec. Scale bar = 10 μm. Video S2: Repair induced by SLO. HeLa cells transfected with ANXA6-YFP (green) were challenged with 250 HU/mL wild-type SLO in the presence of 2 μg/mL TO-PRO3 (blue) and imaged at 37 °C by live cell confocal imaging at 1.5 frames/second. Triton-X-100 was added at the end as a positive control for cell permeabilization. Images were bleach-corrected by histogram matching. Time shows min:sec. Scale bar = 10 μm. Video S3: Repair induced by PFO D434K. HeLa cells transfected with ANXA6-YFP (green) were challenged with 125 HU/mL PFO D434K in the presence of 2 μg/mL TO-PRO3 (blue) and imaged at 37 °C by live cell confocal imaging at 2 frames/second. Triton-X-100 was added at the end as a positive control for cell permeabilization. Images were bleach-corrected by histogram matching. Time shows min:sec. Scale bar = 10 μm. Video S4: Repair induced by PFO D434S. HeLa cells transfected with ANXA6-YFP (green) were challenged with 1000 HU/mL PFO D434S in the presence of 2 μg/mL TO-PRO3 (blue) and imaged at 37 °C by live cell confocal imaging at 2 frames/second. Triton-X-100 was added at the end as a positive control for cell permeabilization. Images were bleach-corrected by histogram matching. Time shows min:sec. Scale bar = 10 μm. Video S5: Repair induced by SLO S505D. HeLa cells transfected with ANXA6-YFP (green) were challenged with 500 HU/mL SLO S505D in the presence of 2 μg/mL TO-PRO3 (blue) and imaged at 37 °C by live cell confocal imaging at 1.5 frames/second. Triton-X-100 was added at the end as a positive control for cell permeabilization. Images were bleach-corrected by histogram matching. Time shows min:sec. Scale bar = 10 μm. Video S6: Monomer-locked SLO does not induce repair. HeLa cells transfected with ANXA6-YFP (green) were challenged with monomer-locked SLO in the presence of 2 μg/mL TO-PRO3 (blue) and imaged at 37 °C by live cell confocal imaging at 1 frame/second. Triton-X-100 was added at the end as a positive control for cell permeabilization. Images were bleach-corrected by histogram matching. Time shows min:sec. Scale bar = 10 μm.
